# Severe Lesions Involving Cortical Cholinergic Pathways Predict Poorer Functional Outcome in Acute Ischemic Stroke

**DOI:** 10.1161/STROKEAHA.118.023196

**Published:** 2018-11-01

**Authors:** Jian-Feng Qu, Yang-Kun Chen, Gen-Pei Luo, Jiang-Hao Zhao, Huo-Hua Zhong, Han-Peng Yin

**Affiliations:** 1 From the Department of Neurology, Dongguan People’s Hospital (Affiliated Dongguan Hospital, South Medical University), Guangdong Province, China (J.-F.Q., Y.-K.C., G.-P.L., J.-H.Z., H.-H.Z., H.-P.Y.); 2 Faculty of Neurology, Guangdong Medical University, Zhanjiang, China (H.-H.Z.).

**Keywords:** activities of daily living, brain ischemia, infarction, magnetic resonance imaging, stroke

## Abstract

**Background and Purpose—:**

The aim of the study was to assess the effect of lesion severity in cortical cholinergic pathways in acute ischemic stroke patients on functional outcomes.

**Methods—:**

The study sample consisted of 214 men (70.9%) and 88 women (29.1%) with acute ischemic stroke. We used the Cholinergic Pathways Hyperintensities Scale (CHIPS) to assess the severity of lesions in cortical cholinergic pathways using brain magnetic resonance imaging. The other magnetic resonance imaging parameters included infarction, white matter lesions, and medial temporal lobe atrophy. Functional outcome was assessed using the Lawton activities of daily living (ADL) scale at 3 and 6 months after the index stroke. We also assessed disability status using the modified Rankin Scale.

**Results—:**

Univariate analysis showed that patients with poor functional outcomes were older, more likely to be men, had a higher National Institutes of Health Stroke Scale (NIHSS) score on admission, and had more frequent histories of previous stroke and infection complications. They also had significantly more frequent cortical infarcts, left subcortical infarcts, a larger infarct volume, more severe medial temporal lobe atrophy, and periventricular hyperintensities, and higher CHIPS scores. In the multiple regression analysis, model 1 showed that age and NIHSS score on admission were significant predictors of poor ADL at 3 months, with an *R*^2^ of 45.4% fitting the model. Age, NIHSS score on admission and stroke subtype were also significant predictors of poor ADL at 6 months, with an *R*^2^ of 37.9% fitting the model. In model 2, sex, previous stroke, NIHSS score on admission, right cortical infarcts, left subcortical infarcts and CHIPS score were significant predictors for poor ADL at 3 months, with an *R*^2^ of 53.5%. NIHSS score on admission, stroke subtype, and CHIPS score were significant predictors for poor ADL at 6 months, with an *R*^2^ of 40.2%. After adjustment for confounders, CHIPS score was also a significant predictor for poor modified Rankin Scale, both at 3 and 6 months. Even after removing patients with moderate-to-severe white matter lesions, higher CHIPS scores still correlated with poorer ADL and modified Rankin Scale both at both 3 and 6 months.

**Conclusions—:**

In patients with acute ischemic stroke, cortical cholinergic pathways impairment is common, and the severity of lesions in the cortical cholinergic pathways may significantly predict a poorer functional outcome.

**Clinical Trial Registration—:**

URL: http://www.chictr.org.cn/index.aspx. Unique identifier: ChiCTR1800014982.

## Abstract 2

Supplemental Digital Content is available in the text.

The mainstay of the cholinergic system in humans consists of a specific group of cells in the basal forebrain, including the diagonal band of Broca, the medial septal nuclei, and the nucleus basalis of Meynert and projecting fibers from these cells.^1,2^ The cortical cholinergic pathways (CCP) represent fibers from the nucleus basalis of Meynert which have been identified to branch into the medial and lateral pathways.^1^ Some studies have found that chronic vascular lesions interrupting the CCP may lead to the depletion of acetylcholine, thus resulting in dementia.^3,4^

However, the CCP are seldom studied in acute ischemic stroke. One previous study, conducted in rats, suggested that middle cerebral artery occlusion resulted in functional disturbances and disruption of the cholinergic pathway between the frontal cortex and the nucleus basalis of Meynert.^5^ A Korean study further found that disruption of cholinergic pathways might contribute to newly developed dementia after acute ischemic stroke.^6^ However, the contribution of CCP impairment to functional status has not been widely studied. The activities of daily living (ADL) scale is often regarded as a functional outcome of acute ischemic stroke. We conducted the current study to explore the relationship between CCP lesions and functional status in patients with acute ischemic stroke.

## Methods

Requests for access to the data and analysis tools in this article will be openly considered. The data that support the findings of this study are available from the corresponding author upon reasonable request.

### Participants and Setting

The study was conducted at Division I, Department of Neurology, Dongguan People’s Hospital between January 1, 2017, and December 30, 2017. The inclusion criteria for the study were (1) aged over 18 years; (2) first or recurrent acute ischemic stroke occurring within 7 days before admission (the diagnosis of acute ischemic stroke was in accordance with the American Heart Association Stroke Council criteria^7^); and (3) a complete brain magnetic resonance imaging (MRI) examination. Exclusion criteria included (1) transient ischemic attack, cerebral hemorrhage, subdural hematoma, or subarachnoid hemorrhage; (2) a lack of a complete set of clinical data (such as no complete brain MRI); (3) death during hospitalization; (4) patients or their relatives refused to sign a consent inform; (5) patients with severe comorbidities (such as malignant tumors, severe organ dysfunction).

The study protocol was approved by the Ethics Committee of Dongguan People’s Hospital. The consent of all subjects was obtained in accordance with the Declaration of Helsinki.

### Demographic Data Collection

The demographic and clinical variables included age, sex, history of stroke, vascular risk factors, neurological deficit status which was assessed using the National Institutes of Health Stroke Scale (NIHSS), infections and treatment. Infection was defined as occurring after 48 hours with a temperature ≥37.3°C after admission according to data from medical records, which was defined as a hospital-acquired infection. Ischemic stroke subtype was judged in accordance with the Trial of ORG 10172 in the Acute Stroke Treatment subtype system by the attending neurologist during hospitalization.^8^

### Follow-Up of the Participants

All participants were followed up for 3 and 6 months after the index stroke via telephone by Dr Qu. Functional status was assessed using the Lawton ADL scale,^9^ which is composed of basic ADL (BADL) and instrumental ADL (IADL). The components of BADL include 6 questions measuring the different levels of ability for toilet activity, feeding, dressing, grooming, physical ambulation, and bathing. The total score is calculated by summing up the points obtained on each item, with a maximum score of 24. The IADL examines a person’s present functional level and identifies improvement or deterioration over time. The 8 domains of function measured with IADL are the ability to use a telephone, shopping, food preparation, housekeeping, laundry, mode of transportation, responsibility for own medications and ability to handle finances. The total IADL score is calculated by summing up the points obtained on each item, and the maximum IADL score is 32. Therefore, the total ADL score ranges from 14 to 56. A higher ADL score reflects poorer ADL performance. We defined a poor functional outcome as a score higher than the 75% quartile of the ADL. At the same time, we also assessed disability at 3 and 6 months according to the modified Rankin Scale (mRS). A poor mRS was defined as ≥3 points. Recurrence of stroke and death during the follow-up period were also recorded.

### MRI Assessment

Brain MRI scanning, including T1-weighted imaging, T2-weighted imaging, and diffusion-weighted imaging, were performed on each participant using a 3.0-T system (Sonata, Siemens Medical, Erlangen, Germany) within 7 days of admission. Diffusion-weighted image spin echo echo-planar imaging; repetition time/echo time/excitation=2162/76/1, matrix=128×128, field of view =230 mm, slice thickness/gap =6/1 mm, echo-planar imaging factor =47, acquisition time =25.9 seconds) with 3 orthogonally applied gradients was used with a b value of 0 and 1000. Axial SE T1 (repetition time/echo time/excitation =488/15/1, field of view =230 mm, slice thickness/gap =6/1 mm, matrix =256×256, time of acquisition =1 minute 24.8 seconds) and TSE T2 (repetition time/echo time/excitation =3992/110/2, turbo factor of 15, field of view =230 mm, slice thickness/gap =6/1 mm, matrix of 512×512, time of acquisition =1 minute 55.8 seconds) images were also acquired.

A neurologist (Dr Luo), who was blinded to the patient’s clinical information and the assessment results, measured the MRI variables as follows:

#### Assessment of Lesions Involving the CCP

**Figure 1.**
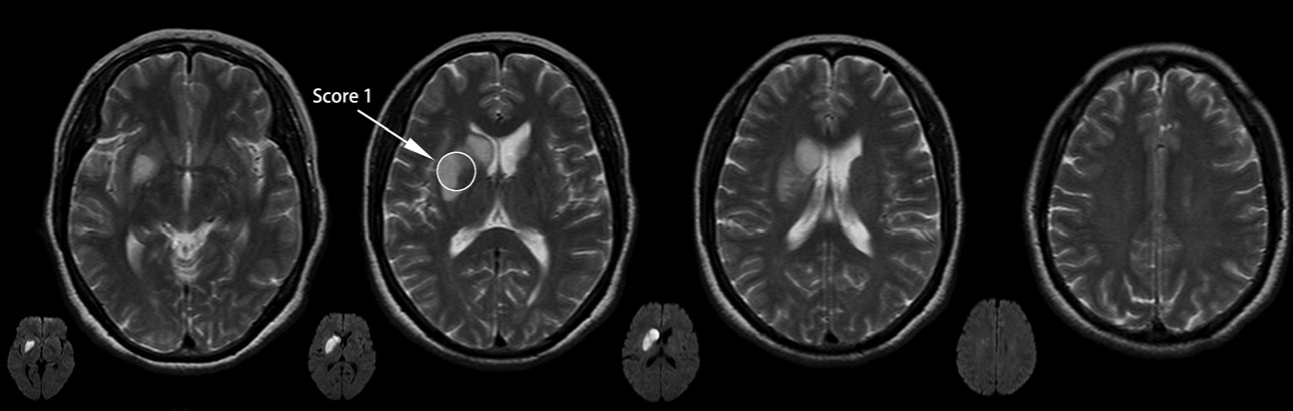
Cholinergic Pathways Hyperintensities Scale (CHIPS) scores from representative patients with acute ischemic stroke.

**Figure 2.**
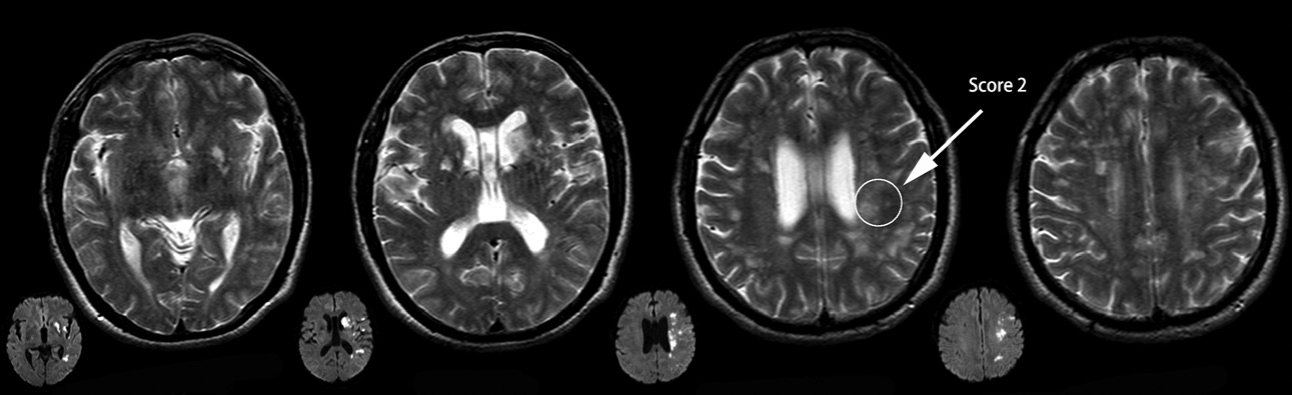
Cholinergic Pathways Hyperintensities Scale (CHIPS) scores from representative patients with both acute ischemic stroke and white matter lesions (WMLs).

Lesions involving the CCP were assessed using the Cholinergic Pathways Hyperintensities Scale (CHIPS).^10^ CHIPS was developed based on immunohistochemical tracings of the cholinergic pathways in humans, superimposed on structural MRI images.^1^ This scale was used to rate the 4 index slices spanning the third and lateral ventricles in the T2 sequence of an axial MRI, which were termed the low external capsule (slice 1), high external capsule (slice 2), corona radiate (slice 3), and centrum semiovale (slice 4). The severity of white matter hyperintensities (WMHs) was visually rated on a 3-point scale for each region (0=normal; 1=mild [<50% of region involved]; 2=moderate to severe [>50% of region involved]). Each slice was weighted to account for the decreasing concentration of cholinergic fibers as they project up and fan out in the whiter matter (maximum weight [4] for slice 1; minimal weight [1] for slice 4). Lesions of the nucleus basal were set at 50 per hemisphere when combining each regional score with the appropriate factor, with a total maximum of 100 per scan.^11^ Lesions involving the CCP mainly included a hyperintensity signal on the MRI-T2 sequence indicating acute infarction (Figure 1), old infarction or WMHs (Figure 2).

#### Site and Volume Assessment of Acute Lesions in the Diffusion-Weighted Imaging Sequence

The sites of acute infarcts were divided into cortical regions and subcortical regions, brain stem, and cerebellum. The cortical lesions included the frontal, temporal, parietal, and occipital lobe, while the subcortical regions included the white matter, basal ganglia, and thalamus. Acute infarcts were defined as areas of restricted water diffusion identified on diffusion-weighted imaging with b values of 1000 together with hypointensity on the corresponding apparent diffusion coefficient map. The total area of acute infarcts on diffusion-weighted imaging was measured using manual outlines. The total volume was calculated by multiplying the total area by the sum of the slice thickness and the gap.

#### White Matter Lesions

The severity of WMLs was graded using the 4-point scale described by Fazekas et al,^12^ which included periventricular hyperintensities and deep WMHs (DWMHs) which were scored on fluid-attenuated inversion recovery images separately.

#### Medial Temporal Lobe Atrophy

Medial temporal lobe atrophy (MTLA) was evaluated using Schelten scale.^13^The rater judged the severity of MTLA on the MRI coronary section based on standard images, ranging from 0 to 4, representing no atrophy to severe atrophy.

Intra-rater reliability tests were performed on 10 stroke patients by the same MRI rater (Dr Luo). The intra-rater agreement of MRI measurements was good to excellent: CHIPS-intraclass coefficient 0.80; volume of infarction-intraclass coefficient 0.85; WMHs—intra-rater κ 0.82; MTLA—intra-rater κ 0.86.

### Statistical Analysis

Statistical analyses were performed using SPSS for Windows (V 24.0, SPSS Inc, Chicago, IL). Descriptive data are presented as proportions, means or medians, as appropriate. A univariate analysis comparing putative risk factors between patients with favorable and poor functional outcome, based on ADL, was performed at 3 and 6 months, respectively. In the logistic regression analysis, we used a backward elimination procedure. The poor outcomes served as dependent variables. Then, risk factors with a value of *P*<0.05 were analyzed through a multivariate logistic regression analysis using a backward stepwise selection strategy. Correlations were conducted to test the collinearity between the candidate independent variables. If the correlation coefficient between any of these putative risk factors was ≥0.40, then variables with a smaller *P* value were entered into the logistic regression. We also analyzed the relationship between the CHIPS score and mRS adjusted for the putative confounders. The odds ratio of an independent risk factor was interpreted as the risk of poor outcome when all other risk factors were held constant. The significance level was set at 0.05 (2-sided).

## Results

Four hundred fifty-six patients with their first or recurrent acute ischemic stroke were consecutively admitted during the study period. Of the 456 consecutive patients, 6 (1.3%) patients died during hospitalization. Patient selection is described in the flowchart (Figure I in the online-only Data Supplement). Finally, 302 patients were included in the analysis. In comparison with the excluded patients, the included patients did not differ significantly in terms of sex (men, 70.9% versus66.2%, *P*=0.311), age (61.3±14.8 versus63.9±12.7, *P*=0.062) or the NIHSS score on admission (4 [2–8] versus3 [2–5.25], *P*=0.148).

The baseline characteristics of the recruited patients are summarized in Table 1. The study sample consisted of 214 men (70.9%) and 88 women (29.1%), with a mean age of 61.3 years (range, 19–91 years). The median NIHSS score on admission was 4 (range, 0–28). Of the 35 cases with infections, 22 were respiratory infections, 9 were urinary infections, 3 were sepsis, and 1 was other type infection.

**Table 1.**
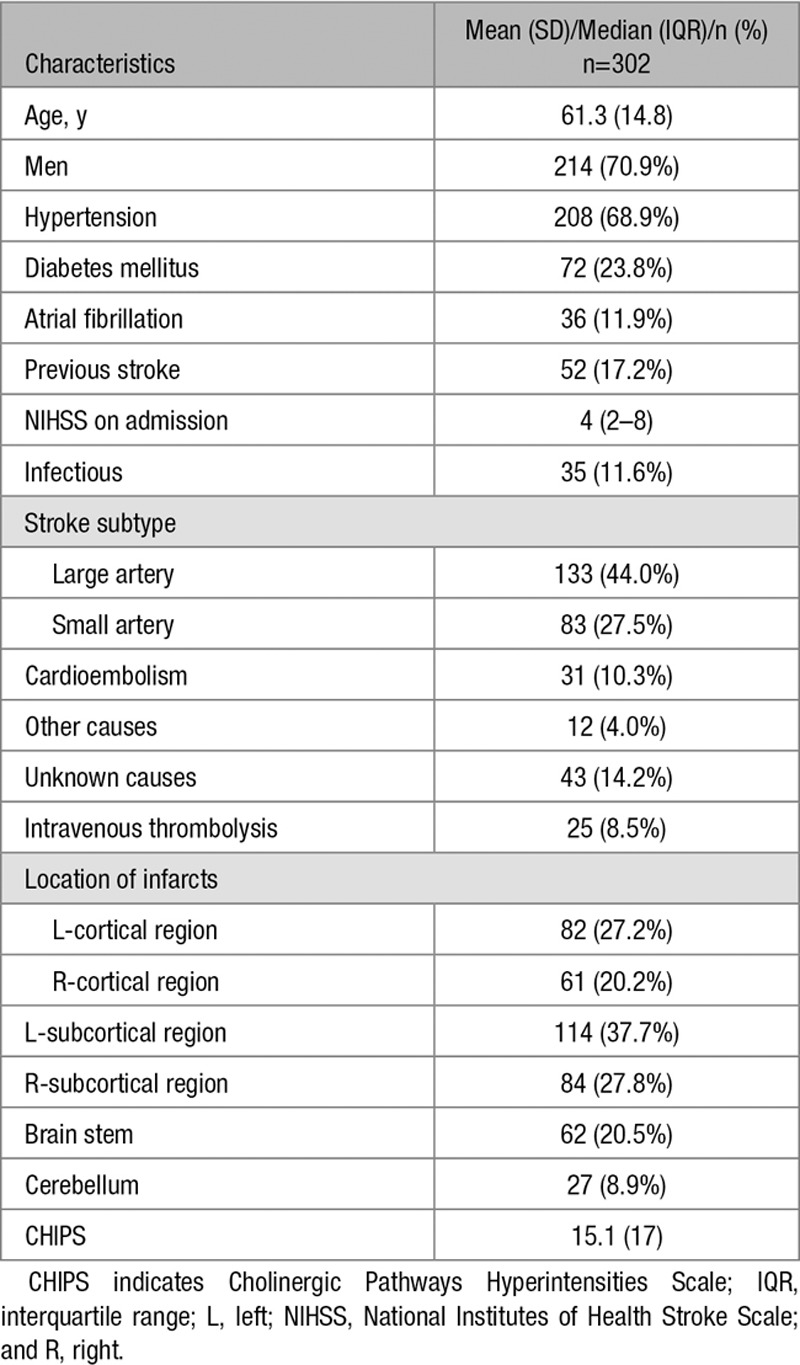
Demographics and Clinical Characteristics of the Study Sample

Subsequently, 2 patients died and 20 patients were lost to follow-up at 3 months, thus, 280 patients were included in the evaluation; and additional 5 patients were lost to follow-up, 275 patients were evaluated at 6 months.

### Univariate Correlates of ADL

In the univariate analysis, patients with poor functional outcomes were older, more likely to be men, had a higher NIHSS score at admission, and had a more frequent history of previous stroke and infection complications. They also had significantly more frequent cortical infarcts, left subcortical infarcts, larger infarct volume, more severe MTLA, and periventricular hyperintensities, and higher CHIPS scores (Table 2).

**Table 2.**
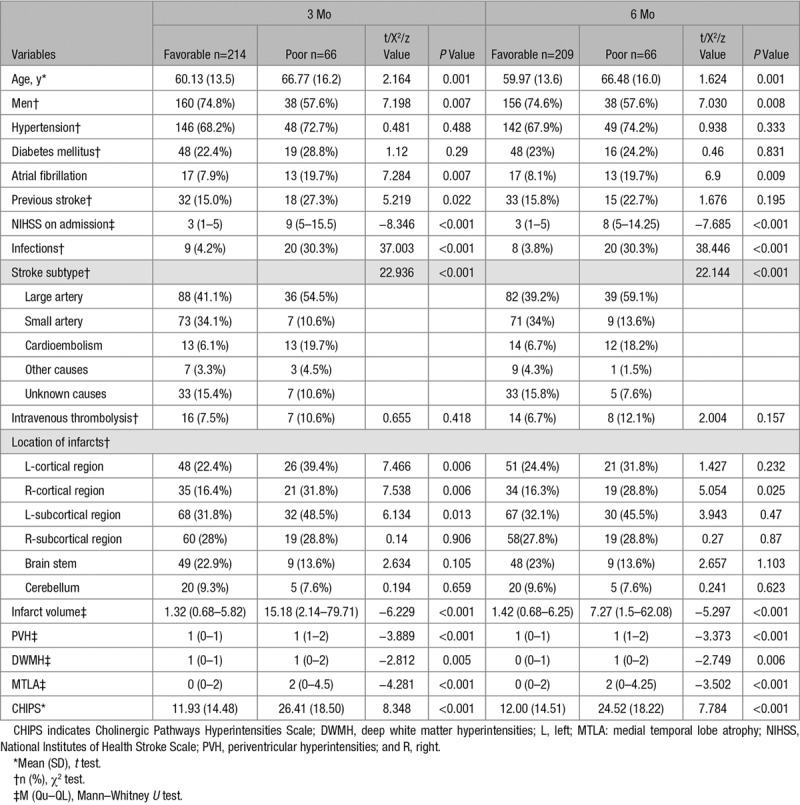
Risk Factors for Poor Outcomes at 3 and 6 Months (Univariable Analysis)

### Multiple Regression Analysis of Functional Outcomes

Multiple stepwise regression models are presented in Table 3. Model 1 only included clinical variables, whereas model 2 included both clinical and MRI variables. In model 1, ADL served as the dependent variable, while age, sex, NIHSS score on admission, atrial fibrillation, previous stroke, and stroke subtype were independent variables of the model at 3 months. Infection was not included in the model as it was highly correlated with NIHSS score on admission (*r*=0.507). Age and NIHSS score at admission were significant predictors of poor ADL at 3 months, with an *R*^2^ of 45.4% fitting the model. Age, NIHSS score on admission and stroke subtype were also significant predictors of poor ADL at 6 months, with an *R*^2^ of 37.9% fitting the model.

**Table 3.**
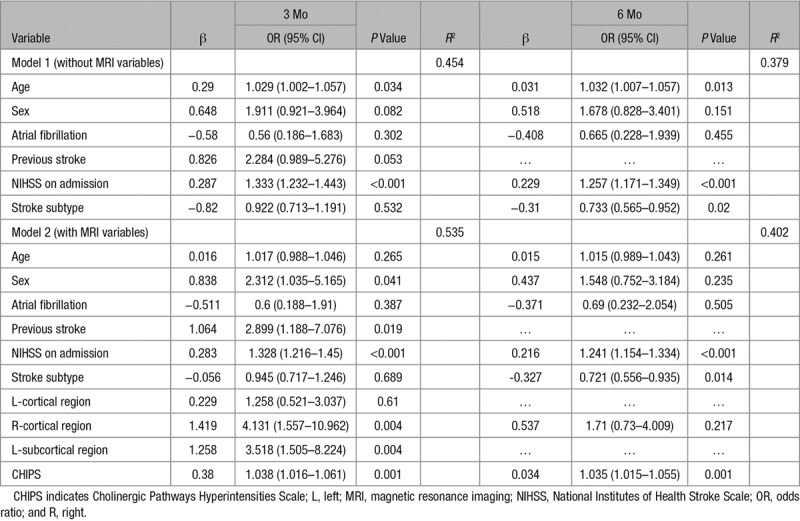
Multivariate Logistic Regression of Risk Factors for Poor ADL

In model 2, left cortical, right cortical, left subcortical region infarct, and CHIPS were also entered into the logistic regression model. Periventricular hyperintensities and DWMH were not included in the model as they were highly correlated with CHIPS score (*r*=0.67 and 0.697, respectively). Infarct volume was not included in the model as it was highly correlated with NIHSS at admission (*r*=0.534). MTLA was not included in the model as it was highly correlated with age (*r*=0.558). Sex, previous stroke, NIHSS score on admission right cortical infarcts, left subcortical infarcts and CHIPS score were significant predictors for poor ADL at 3 months, with an *R*^2^ of 53.5%. NIHSS score on admission, stroke subtype, and CHIPS score were significant predictors for poor ADL at 6 months, with an *R*^2^ of 40.2% (Table 3). After adjustment for confounders, CHIPS score was identified as a significant predictor for poor mRS, both at 3 and 6 months (Table 4).

**Table 4.**
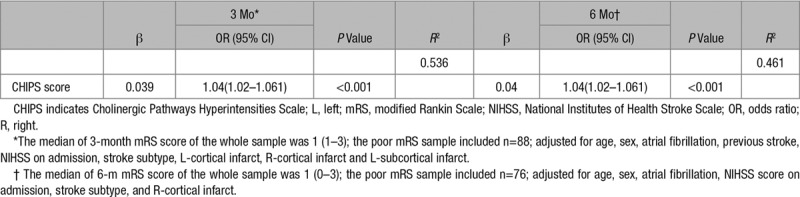
Multivariate Logistic Regression of Poor mRS According to CHIPS Score

As CHIPS score was significantly correlated with periventricular hyperintensities and DWMH, this suggested that moderate-severe WMLs might confound the accuracy of acute ischemic stroke analysis involving the CCP. Therefore, we conducted another logistic regression model without patients with moderate-severe WMLs for ADL (Table I in the online-only Data Supplement) and mRS (Table II in the online-only Data Supplement), respectively. The results showed that even after adjusting for moderate-severe WMLs, CHIPS score was still a significant predictor for poor ADL and mRS at 3 and 6 months, respectively.

## Discussion

In this longitudinal observational study, we first assessed the associations between lesions involving CCP and functional outcome in Chinese patients with acute ischemic stroke. Our main finding was that more severe CCP lesions were predictive of poorer functional outcome at 3 and 6 months after stroke, even after adjusting for possible confounding factors.

The CHIPS was initially introduced to evaluate WMHs affecting the CCP in patients with Alzheimer disease.^10^ The CHIPS total score showed good correlation with lesion volume within the cholinergic pathways (Spearman=0.87, *P*<0.0001). Higher CHIPS scores suggest more severe CCP impairment.^10^ However, in patients with acute ischemic stroke, not only WMLs but also acute and old infarctions would affect the CCP. Therefore, it might be reasonable to take infarctions into consideration as well when calculating the CHIPS score.

The current study suggests that impairment of CCP correlated with poor functional outcome assessed by ADL at 3 and 6 months after stroke. Losses in CCP can be seen in Alzheimer disease,^11^ vascular dementia,^14^ and Parkinson disease,^15^ which correlate with cognitive dysfunction. A Korean study revealed that impairment of cholinergic pathways might contribute to newly developed dementia after acute ischemic stroke.^6^ To the best of our knowledge, complete ADL requires not only dependent BADL but also memory function, and satisfactory executive^16^ and visuospatial functions.^17^ Cholinergic pathway lesions are thought to be closely related to executive function, attention, memory, and cognitive decline.^18^A previous study demonstrated that cortical cholinergic integrity plays a specific role in executing top-down control to resist external distraction,^19^ while thalamic-cholinergic innervation plays an important role in bottom-up, stimulus-driven attention, and target detection.^20^ We hypothesized that impaired ADL may be caused by cognitive impairment related to the impairment of CCP.^18^

Surprisingly, we also found that CCP impairment also significantly correlated with poor mRS. As we know, the mRS mainly reflects the overall degree of disability, including both physical function and functional prognosis.^21^ Cognitive status has some impact on these daily functions. CCP is closely related to cognitive function and may also affect the degree of disability. CHIPS can be measured conveniently in clinical practice and has a certain value in guiding and predicting the prognosis of patients.

The advantages of our study are as follows: (1) this was a consecutively recruited and prospective study and (2) we used comprehensive neuroimaging parameters, which include CCP hyperintensities, infarction, WMLs, and MTLA. There are also some limitations to the current study that need to be considered. First, the sample size was relatively small. Second, we did not evaluate cognitive status before and during hospitalization, which might be a mediator between CCP impairment and functional outcomes. Third, we did not assess the NIHSS at follow-up after stroke, leading to an inadequate evaluation of residual neurological deficits poststroke.

In conclusion, CCP lesion severity might predict a poorer functional outcome in patients with acute ischemic stroke. Further prospective studies with a larger sample size and longer follow-up period are now warranted to clarify the link between CCP impairment and ADL performance.

## Sources of Funding

This study was supported by the Medical Scientific Research Foundation of Guangdong Province, China (grant no. B2017054).

## Disclosures

None.

## Supplementary Material

## References

[1] Selden NR, Gitelman DR, Salamon-Murayama N, Parrish TB, Mesulam MM (1998). Trajectories of cholinergic pathways within the cerebral hemispheres of the human brain.. Brain.

[2] Mesulam MM (2004). The cholinergic innervation of the human cerebral cortex.. Prog Brain Res.

[3] Mesulam M, Siddique T, Cohen B (2003). Cholinergic denervation in a pure multi-infarct state: observations on CADASIL.. Neurology.

[4] Kim HJ, Moon WJ, Han SH (2013). Differential cholinergic pathway involvement in Alzheimer’s disease and subcortical ischemic vascular dementia.. J Alzheimers Dis.

[5] Kataoka K, Hayakawa T, Kuroda R, Yuguchi T, Yamada K (1991). Cholinergic deafferentation after focal cerebral infarct in rats.. Stroke.

[6] Lim JS, Kim N, Jang MU, Han MK, Kim S, Baek MJ (2014). Cortical hubs and subcortical cholinergic pathways as neural substrates of poststroke dementia.. Stroke.

[7] Jauch EC, Saver JL, Adams HP, Bruno A, Connors JJ, Demaerschalk BM, American Heart Association Stroke Council; Council on Cardiovascular Nursing; Council on Peripheral Vascular Disease; Council on Clinical Cardiology (2013). Guidelines for the early management of patients with acute ischemic stroke: a guideline for healthcare professionals from the American Heart Association/American Stroke Association.. Stroke.

[8] Adams HP, Bendixen BH, Kappelle LJ, Biller J, Love BB, Gordon DL (1993). Classification of subtype of acute ischemic stroke. Definitions for use in a multicenter clinical trial. TOAST. Trial of Org 10172 in Acute Stroke Treatment.. Stroke.

[9] Lawton MP, Brody EM (1969). Assessment of older people: self-maintaining and instrumental activities of daily living.. Gerontologist.

[10] Bocti C, Swartz RH, Gao FQ, Sahlas DJ, Behl P, Black SE (2005). A new visual rating scale to assess strategic white matter hyperintensities within cholinergic pathways in dementia.. Stroke.

[11] Fukui T, Hieda S, Bocti C (2006). Do lesions involving the cortical cholinergic pathways help or hinder efficacy of donepezil in patients with Alzheimer’s disease?. Dement Geriatr Cogn Disord.

[12] Fazekas F, Kleinert R, Offenbacher H, Schmidt R, Kleinert G, Payer F (1993). Pathologic correlates of incidental MRI white matter signal hyperintensities.. Neurology.

[13] Scheltens P, Leys D, Barkhof F, Huglo D, Weinstein HC, Vermersch P (1992). Atrophy of medial temporal lobes on MRI in “probable” Alzheimer’s disease and normal ageing: diagnostic value and neuropsychological correlates.. J Neurol Neurosurg Psychiatry.

[14] Kim HJ, Moon WJ, Han SH (2013). Differential cholinergic pathway involvement in Alzheimer’s disease and subcortical ischemic vascular dementia.. J Alzheimers Dis.

[15] Park HE, Park IS, Oh YS, Yang DW, Lee KS, Choi HS (2015). Subcortical whiter matter hyperintensities within the cholinergic pathways of patients with dementia and parkinsonism.. J Neurol Sci.

[16] Fukui T, Lee E (2009). Visuospatial function is a significant contributor to functional status in patients with Alzheimer’s disease.. Am J Alzheimers Dis Other Demen.

[17] Boyle PA, Paul RH, Moser DJ, Cohen RA (2004). Executive impairments predict functional declines in vascular dementia.. Clin Neuropsychol.

[18] Chen YK, Xiao WM, Li WY, Liu YL, Li W, Qu JF (2015). Neuroimaging indicators of the performance of instrumental activities of daily living in Alzheimer’s disease combined with cerebrovascular disease.. Geriatr Gerontol Int.

[19] Kim K, Müller MLTM, Bohnen NI, Sarter M, Lustig C The cortical cholinergic system contributes to the top-down control of distraction: evidence from patients with Parkinson’s disease [published online December19, 2017].. Neuroimage.

[20] Kim K, Müller MLTM, Bohnen NI, Sarter M, Lustig C (2017). Thalamic cholinergic innervation makes a specific bottom-up contribution to signal detection: evidence from Parkinson’s disease patients with defined cholinergic losses.. Neuroimage.

[21] van Swieten JC, Koudstaal PJ, Visser MC, Schouten HJ, van Gijn J (1988). Interobserver agreement for the assessment of handicap in stroke patients.. Stroke.

