# Supplementary material for: Severe Lesions Involving Cortical Cholinergic Pathways Predict Poorer Functional Outcome in Acute Ischemic Stroke
Source: Stroke. 2018 Nov 1;49(12):2983–9. doi: 10.1161/STROKEAHA.118.023196 (PMC6257508; doi:10.1161/STROKEAHA.118.023196)
Supplement: Supplementary file 1 [file str-49-2983-s001.pdf]

SUPPLEMENTAL MATERIAL

Severe lesions involving cortical cholinergic pathways predict poorer functional outcome in acute ischemic stroke

Supplemental contents

Supplemental Figure Ⅰ. Flow-chart.

Supplemental Table Ⅰ. Multivariate logistic regression of poor ADL according to CHIPS score without moderate-severe white matter lesions.

Supplemental Table Ⅱ. Multivariate logistic regression of poor mRS according to CHIPS score without moderate-severe white matter lesions.

Supplemental Figure Ⅰ. Flow-chart.

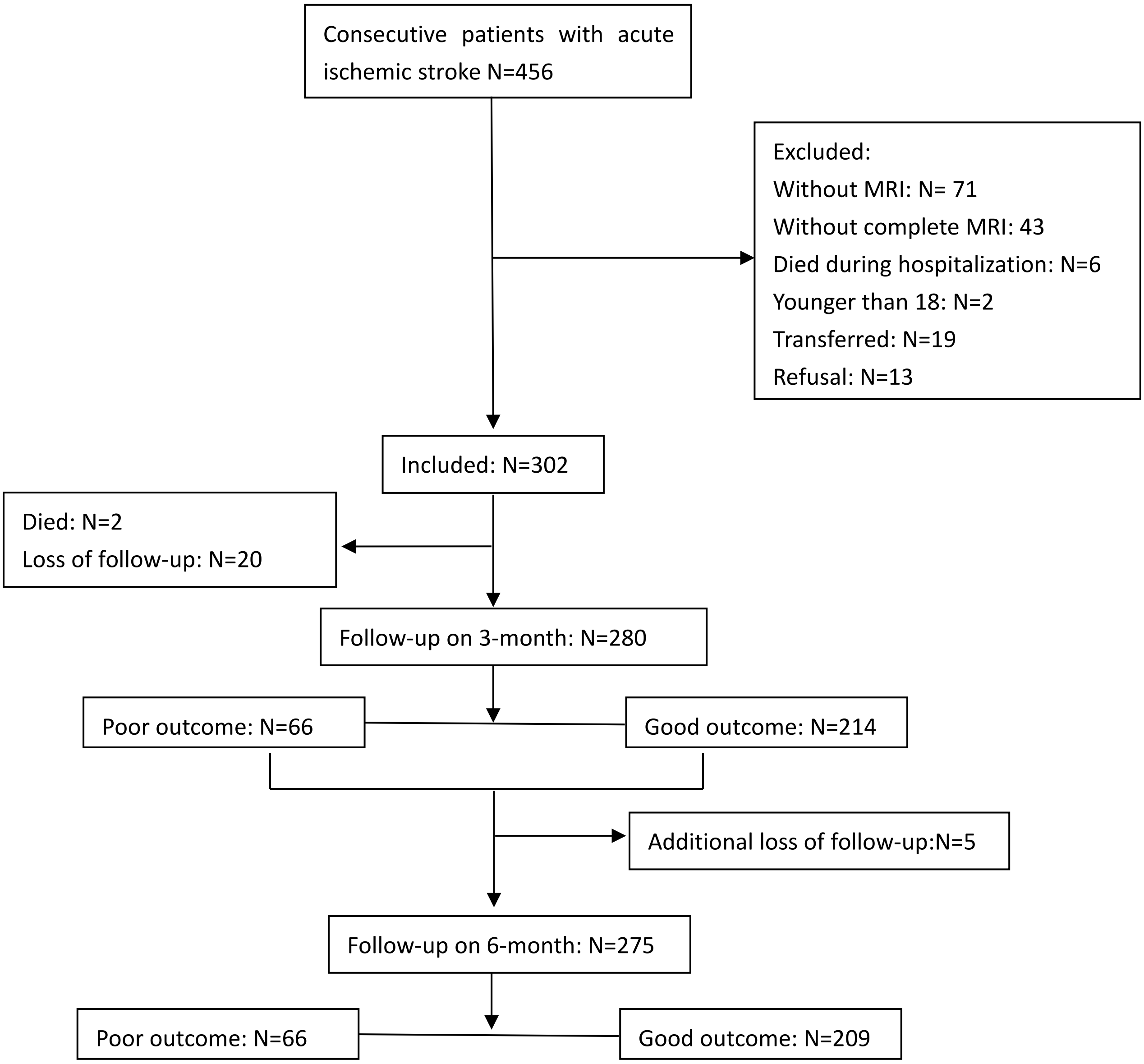

Supplemental Table Ⅰ. Multivariate logistic regression of poor ADL according to CHIPS score without moderate-severe white matter lesions.[a]

Variable	3-months[b]				6-months[c]			
	β	*OR* (95% *CI*)	*P* value	R^2	*β*	*OR* (95% *CI*)	*P* value	R^2
				0.566				0.438
CHIPS	0.066	1.068(1.020–1.119)	0.005		0.059	1.061(1.018–1.107)	0.006	

Note: [a] moderate-severe WML was judged as total score of PVH and DWMH >3 points. [b] n=206, loss to follow-up was 16, n=190 were analyzed, the poor ADL sample included n=34; adjusted for age, gender, atrial fibrillation, previous stroke, NIHSS score on admission, stroke subtype, L-cortical infarct, R-cortical infarct and L-subcortical infarct. [c] n=206, loss to follow-up was 18, n=188 were analyzed, the poor ADL sample included, n = 35; adjusted for age, gender, atrial fibrillation, NIHSS score on admission, stroke subtype, and R-cortical infarct.

Supplemental Table Ⅱ. Multivariate logistic regression of poor mRS according to CHIPS score without moderate-severe white matter lesions.

Variable	3-months[a]				6-months[b]			
	β	*OR* (95% *CI*)	*P* value	R^2	*β*	*OR* (95% *CI*)	*P* value	R^2
				0.618				0.483
CHIPS	0.081	1.085(1.034–1.138)	0.001		0.064	1.066(1.023–1.112)	0.003	

Note: [a] The poor mRS sample included n=51; adjusted for age, gender, atrial fibrillation, previous stroke, NIHSS score on admission, stroke subtype, L-cortical infarct, R-cortical infarct and L-subcortical infarct. [b] The poor mRS sample included, n = 43; adjusted for age, gender, atrial fibrillation, NIHSS score on admission, stroke subtype, and R-cortical infarct.
